# The Efficacy of Buprenorphine in Preoperative and Postoperative Patients: A Literature Review

**DOI:** 10.7759/cureus.60341

**Published:** 2024-05-15

**Authors:** Keyur Patel, Paul Lee, Jessica Witherspoon, Kunnal Patel, Richard Jermyn

**Affiliations:** 1 College of Medicine, NeuroMusculoskeletal Institute, Rowan-Virtua School of Osteopathic Medicine, Stratford, USA; 2 Physical Medicine and Rehabilitation, NeuroMusculoskeletal Institute, Rowan-Virtua School of Osteopathic Medicine, Stratford, USA

**Keywords:** buprenorphine, post op pain management, post-acute care, pre-operative management, pre operative evaluation, acute pain

## Abstract

Although research suggests that less than half of individuals who have surgical procedures report effective postoperative pain alleviation, the majority of patients endure acute postoperative discomfort. To lessen and manage postoperative pain, a variety of preoperative, intraoperative, and postoperative treatments and management methods are available. For several years an opioid called buprenorphine has become an effective tool to treat opioid use disorder (OUD) in patients across many different demographics. It has however endured barriers to its usage which can be seen when treating patients with chronic pain or postoperative pain, who also have an OUD. While buprenorphine may be underutilized within the clinical setting, the significantly low rates of chronic abuse when using the drug allow it to be an attractive treatment option for patients.

This paper aims to explore a wide range of studies that examine buprenorphine as an analgesic and how it can be used for preoperative pain and postoperative pain. This paper will give an in-depth analysis of buprenorphine and its use in patients with chronic pain as well as OUD.

A systematic literature review was performed by identifying studies through the database PubMed. The data from various publications were gathered with preference being given to publications within the last three years. We reviewed studies that examined the pain level of the patients after having buprenorphine.

Despite long-available pharmacologic evidence and clinical research, buprenorphine has maintained a mystique as an analgesic. Its usage in the treatment of OUD was further influenced by its well-known safety benefits and relative lack of psychomimetic side effects compared to other opioids. For patients accustomed to long-term, high-dose opioids who may be experiencing hyperalgesia but have not been informed about this phenomenon by their doctors or the potential for buprenorphine to resolve it, buprenorphine's pronounced antihyperalgesic effect is a compelling pharmacologic characteristic that makes it particularly attractive as an option.

When used in pre-, peri-, and postoperative circumstances, buprenorphine provides various pain-management benefits and patients can still benefit from effective pain management from mu-opioid agonists while remaining on buprenorphine. Buprenorphine can be continued at a reduced dose as needed to avoid withdrawal symptoms and to improve the analgesic efficiency of mu-opioid agonists used in combination with acute postoperative pain in light of the evidence at hand. Buprenorphine administration needs a patient-centered, multidisciplinary strategy that considers the benefits and drawbacks of the many perioperative therapy options to have the best chance of success.

## Introduction and background

**Figure 1:**
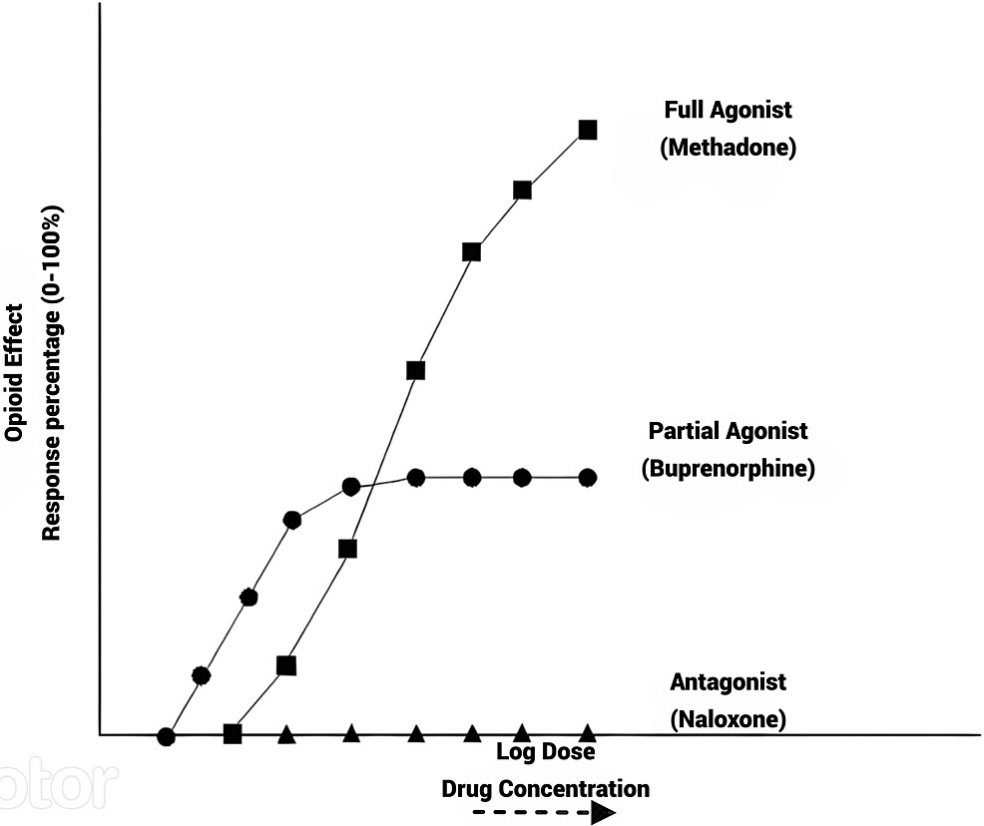
Conceptual representation of opioid effect versus log dose for opioid full agonists, partial agonists, and antagonists Image credit: Reference [2]

Buprenorphine has a distinctive and complicated pharmacological profile and is an effective therapy for opioid dependence [1]. Buprenorphine is a partial mu-opioid agonist with low intrinsic activity and strong receptor affinity, according to preclinical research. Although it also functions as a kappa opioid antagonist, its effects on the mu-receptor seem to be the primary if not the only source of its therapeutic advantages. The maximum effect that buprenorphine can have as a partial mu-opioid agonist will always be less than what a full mu-opioid agonist can have [2]. Furthermore, compared to an opioid agonist with higher intrinsic activity like morphine, buprenorphine requires the occupancy of a greater proportion of receptors to exert a given effect (Figure 1) [2]. Buprenorphine has a high affinity for the receptor, which increases the possibility that it will be bound as opposed to unbound and makes it challenging to remove from the receptor [3]. This complicated pharmacology is expected to support buprenorphine's safety, clinical usage protocol, regulatory control, accessibility, and patient acceptance and compliance in addition to supporting its efficacy. Therefore, knowing buprenorphine's pharmacology and pharmacodynamic effects, particularly in humans, will improve the drug's effectiveness in treating opioid dependence along with pain management.

The efficacy of buprenorphine has been the subject of some of this research, which is discussed elsewhere in this issue. In the clinical laboratory, where some degree of experimental control is acquired over the numerous variables that may interact with the direct pharmacologic effects of buprenorphine, other significant research concerns have been addressed [3].

In a parenteral formulation, buprenorphine was initially approved for clinical use as an analgesic for acute and postoperative pain. Buprenorphine is on the market as a pain reliever in both injectable and sublingual forms [4]. In order to treat subacute or chronic pain, buprenorphine was later developed as a skin patch (brand name: Butrans patch) with a one-week duration of effect. A buccal film for the treatment of pain recently received U.S. approval. This indication is especially important for people who have opioid use disorder (OUD) and concomitant pain because buprenorphine may be effective in treating both conditions. For patients who need opioid analgesia but also show indicators of opioid misuse, the patch or buccal film are good options [4].

Additionally, it has anti-hyperalgesia qualities. Over a dose range of 0.05-0.6 mg, studies have demonstrated a ceiling impact for respiratory depression but not for analgesia. Additionally, it has less impact on GI transit delays, which results in less vomiting and constipation. Other opioids can be utilized for breakthrough pain in the presence of buprenorphine because it does not cause chronic mu-receptor binding. It can be given at regular doses to older patients with renal impairment because the liver is the primary site of metabolism and excretion, and studies have shown that mild-to-moderate liver impairment does not affect the drug's pharmacokinetics [5].

Methods

Search Term Strategy

On December 10, 2022, we identified studies by searching through the database PubMed. The following string of search terms was used to identify peer-reviewed articles in each database: "Buprenorphine" AND "Postoperative" AND “Pain” OR "Management OR “Accessibility” OR “Preoperative” OR “Route of entry” OR “age”, OR “gender”. The individual references were divided into themes. Still, there was a lot of overlap in the articles. Articles were reviewed by Keyur Patel, Paul Lee, and Jessica Witherspoon.

Inclusion criteria

Types of Studies

Most studies used were cross-sectional studies. There were meta-analyses and systematic review papers as well. We also included causality-targeted experimental randomized control studies.

Types of Participants

Most research focused on populations that came from a wide variety of demographic backgrounds. There were some studies that had overlap with the adolescent population. All racial backgrounds were incorporated into the study.

Types of Interventions

We did not want to limit this study to a particular intervention type to obtain as much valuable data as possible on the risk factors we chose to explore. Therefore, the selected studies included the following intervention types: self-reported surveys, physical measurements, electronic health records, and minimal interventions.

Types of Outcome Measures

We reviewed studies that examined the pain level of the patients after having buprenorphine. Many of these studies compared and looked at the prognosis of the patients and measured the patient’s clinical opioid withdrawal scale along with a visual analog scale (VAS) and associated symptoms related to the medications taken.

Data extraction

**Figure 2:**
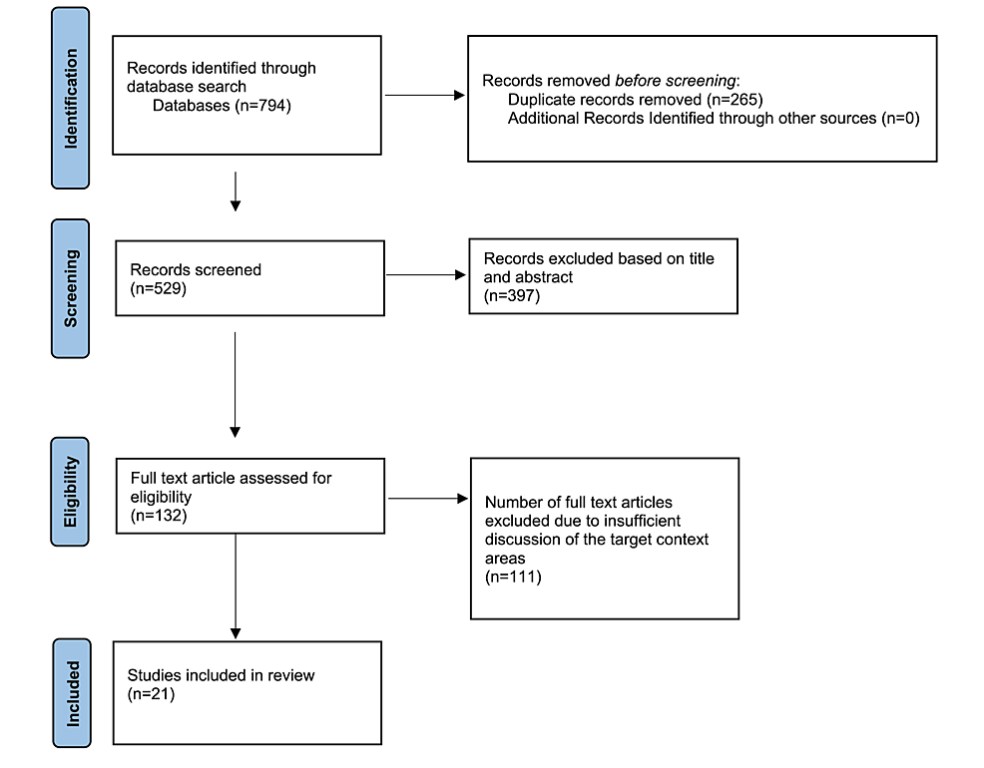
PRISMA flow sheet indicates the studies identified, screened, and included in the review article PRISMA: Preferred Reporting Items for Systematic Reviews and Meta-Analyses

The information, methodology, and findings from the limited selection of papers were extracted. We specifically gathered data on the article's title, authors, and publication year. Sample size, research design, study demographics, and constraints, as well as qualitative and quantitative analysis of results, were all included in the data. As seen in Figure 2 we used the database search and screened the records. Afterward, we looked at the eligible articles and included the remainder in our study.

## Review

Perioperative management of buprenorphine

Care of patients undergoing opioid addiction therapy during surgery provides special challenges for anesthesiologists. Effective postoperative pain management, better patient outcomes, and cost-effective healthcare are the three main objectives of pain management in this patient population. Multimodal analgesics, such as intravenous acetaminophen, gabapentinoids, and low-dose ketamine infusions, have been utilized to lessen the need for postoperative opioid painkillers and to improve postoperative pain. Special considerations must be made for patients receiving methadone and buprenorphine as part of a long-term opioid management program [6]. A possible option includes patients not taking buprenorphine 72 hours before the operation. Due to buprenorphine's partial agonist effect, this removes the partial blockage it had previously created. If a patient is undergoing surgery with moderate to severe pain and is taking more than 16 mg of buprenorphine daily, titrating down to 16 mg allows for greater pain control in the operating room. On the day of surgery, maintain a dosage of 8 mg of buprenorphine. After the surgical pain subsides, taper off the opioid agonist and resume the home buprenorphine dose. Buprenorphine is gradually tapered off over two to three weeks, decreasing by two milligrams per day every two to three days. Buprenorphine can also be rapidly reduced over three days, but the likelihood of relapse is increased [6]. Also, buprenorphine induction treatment can be started in-patient after acute pain has subsided, or the patient can be discharged home on pure opioid agonist medications with a buprenorphine induction process started after discharge.

Opioid agonists are used to alleviate acute pain while buprenorphine maintenance medication is continued. Since buprenorphine competes with opioid agonists for mu-receptor sites, higher doses of these drugs may be required to provide the desired analgesia. Opioids with greater intrinsic mu-activity, such as morphine, fentanyl, or hydromorphone, should be added; in contrast, opioids with lower mu-receptor efficacy, such as codeine and hydrocodone, should be avoided [6].

Buprenorphine maintenance does not result in poorer clinical outcomes after surgery. While being kept on buprenorphine, patients can still receive effective pain relief from mu-opioid agonists. It is advised to continue buprenorphine at a lower dose as necessary to prevent withdrawal symptoms and to enhance the analgesic effectiveness of mu-opioid agonists used in combination with acute postoperative pain [7]. If the patient has an OUD, then the pain management can change. Buprenorphine shouldn't be routinely stopped in the perioperative setting to lower the chance of OUD recurrence. To reduce the risk of opioid recurrence and overdose death in untreated patients with OUD and acute pain, buprenorphine can be started in the perioperative context [8]. Due to tolerance and elevated pain sensitivity, patients with OUD may also need greater doses of opioid analgesics [9].

Preoperative considerations

It is advised that these individuals be assessed before surgery to collect a thorough pain history, perform a physical exam, and check for any further psychiatric or medical comorbidities [10]. Prior to surgery, it is important to assess the patient's preoperative pain, addiction history, and last dose of buprenorphine/naloxone. Because it can assist with opioid dosing, prior experience with surgery and buprenorphine/naloxone should also be considered. Patients may require higher doses of opioids preoperatively and during surgery if buprenorphine/naloxone has been discontinued for more than five days and shifted to pure agonists [11].

A study discovered significant rates of perioperative buprenorphine dose holds using patients who underwent an inpatient surgery in 2018 who were given buprenorphine for OUD. Initiates were performed so patients with OUD receive the correct therapy, as keeping buprenorphine perioperatively is not in line with new clinical recommendations and entails considerable risks [12].

In other studies, preoperative pain management with buprenorphine leads to a lower amount of pain in a postoperative setting. In one specific scenario, it is effective in lumbar discectomy. Seventy-eight patients who were chosen for lumbar discectomy surgery participated in a randomized clinical trial investigation. The patients were split into two groups of 39 each at random. One hour prior to surgery, patients in the buprenorphine and placebo group got 2 mg of sublingual buprenorphine and a placebo. At one, six, and 12 hours, the buprenorphine group's pain ratings significantly differed from the placebo group's (P < 0.005). More analgesics were used in the control group than in the buprenorphine group. The incidence of nausea in the buprenorphine group was considerably lower than in the control group in the first one to six hours following surgery (P <0.05). However, this change was not seen at 12 or 24 h, p > 0.05. The incidence of adverse effects (nausea, vomiting, and pruritus) was not significantly different between the two groups (P > 0.05) [13].

Postoperative considerations

Early mobilization is one of the largest risk factors that lowers morbidity during most procedures. Pain management must be managed to accomplish mobilization. One investigation of arthroscopic knee surgery found buprenorphine as a preferable postoperative analgesic due to its prolonged duration of action. In one trial, buprenorphine and dexmedetomidine were administered intraarticularly. Patients taking intraarticular buprenorphine experienced a considerably longer time until their initial rescue analgesia. Differences in the VAS scores at rest were comparable between the groups, but at the 12th and 24th hours, VAS scores with intraarticular buprenorphine were considerably lower than those with intraarticular dexmedetomidine. At the first, second, and fourth hours, VAS scores on ambulation were equivalent, but at the 8th, 12th, and 24th hours, dexmedetomidine values were considerably higher than those for buprenorphine [14].

A separate study utilized opioid-dependent users who were undergoing laparotomy due to acute abdomen pain and compared the usage of morphine 5 mg IV or buprenorphine 2mg sublingual. They compared the VAS of both groups and the clinical opioid withdrawal score (COWS). At hours 6 and 24, the buprenorphine group's VAS was 4.47 ± 0.73 and 2.67 ± 0.53, respectively. In the morphine group, the equivalent scores were 5.88 ± 0.69 and 4.59 ± 0.74. Patients taking buprenorphine simultaneously reported less severe withdrawal symptoms. The treatment of pain endured by patients who are reliant on opioids is made more difficult by increased pain sensitivity and the emergence of opioid tolerance. The current study recommends sublingual buprenorphine for patients with opioid dependence to manage pain and withdrawal symptoms [15].

Another trial used a double-blind design, to analyze a laparoscopic cholecystectomy versus a single diclofenac patch for postoperative discomfort. The night before surgery, a buprenorphine 10 mg patch was applied. The operative schedule for the following day was decided upon on the previous day's afternoon, and the patch was applied in the evening, leaving enough time until the patient was scheduled to be placed on the operating table at 11 a.m. The patch was preserved for seven days. Preoperatively, a 100 mg diclofenac patch was put on the left or right flank of the same patient, and it was changed after 24 hours. The second group received a placebo patch that was applied to the upper third of the lateral arm (on any side) the evening before surgery and was worn for seven days. Prior to surgery, a 100 mg diclofenac patch was inserted on the left or right lateral flank and removed after 24 hours [16]. In the end, it was statistically significant that the placebo group needed more rescue analgesia than the buprenorphine plus diclofenac patch group did. In postoperative laparoscopic cholecystectomy patients, multimodal analgesia with transdermal buprenorphine (TDB) and diclofenac can be a novel yet straightforward alternative to injectable opioids while delivering effective analgesia [16].

Transdermal opioids are a more recent method of pain management. The purpose of the study was to assess the effectiveness of analgesia using buprenorphine patches (10, 20μg·h-1) and fentanyl patches (25μg·h-1) for pain reduction in patients having arthroscopic lower limb procedures. Fentanyl patches were given to Group 1 (25 μg·h-1), buprenorphine patches to Groups 2 and 3, and buprenorphine patches to Group 3. Groups of the 175 patients were formed [17]. The postoperative pain was assessed using the numerical rating scale (NRS). In Group 3, the mean NRS value was found to be considerably lower throughout a 24-hour period. The least amount of the rescue analgesic diclofenac was utilized overall in Group 3 compared to the other two groups. For the first 24 hours postoperatively, Groups 1 and 2 were found to have considerably higher mean blood pressure and heart rate values than Group 3, but no significant difference was identified after that. Overall, the study demonstrated that buprenorphine patch 20 μg·h-1 was superior to buprenorphine patch 10μg·h-1 and fentanyl patch 25μg·h-1 for postoperative pain following lower limb arthroscopic operations, with no increased hemodynamic instability or negative side effects [17].

When compared to oral tramadol (OT) in a study following proximal femur surgeries, on all seven days beginning 24 hours after surgery, TDB Group members reported considerably lower levels of resting pain and pain with movement. In comparison to the OT Group, the TDB Group had a much lower need for rescue analgesics. In the OT Group, all the patients required rescue analgesia, whereas 68% of the patients in the TDB Group required it. Vomiting was less frequent and satisfaction levels were significantly greater in the TDB Group compared to the OT Group (79% vs. 66%, P< 0.001). When compared to OT, TDB is less likely to cause side effects and is more effective at reducing postoperative pain after 24 hours. It can be used safely for postoperative analgesia [18].

Another study compared the effectiveness of epidural buprenorphine with epidural butorphanol in treating postoperative pain after laparoscopic hysterectomies. Butorphanol is a partial agonist and competitive opioid antagonist. Through the epidural catheter, 1 ml (0.3 mg) of buprenorphine and 1 ml (1 mg) of butorphanol tartrate, both diluted to 10 ml with normal saline, were administered to Group A and Group B, respectively. Visual analog pain scales (VAPSs) were measured every hour up until the sixth hour, and then every two hours until the 12-hour mark. When compared to butorphanol tartrate, buprenorphine’s analgesic effect lasted longer (586.17 ± 73.64 vs. 342.53 ± 47.42 (P<0.001)). Buprenorphine caused more nausea, vomiting (13% vs. 10%), and headaches (20% vs. 13%). In comparison to butorphanol, epidural buprenorphine provided postoperative patients with much less pain and better analgesia with a longer duration of action [19].

Summary of findings

Ultimately, continuing buprenorphine on patients who were placed in for operations lowers the postoperative complications. Additionally, buprenorphine has many pain management properties in pre-, peri-, and postoperative situations. Buprenorphine maintenance does not result in poorer clinical outcomes after surgery. While being kept on buprenorphine, patients can still receive effective pain relief from mu-opioid agonists. In light of the information at hand, we advise continuing buprenorphine at a lower dose as necessary in order to prevent withdrawal symptoms and enhance the analgesic effectiveness of mu-opioid agonists used in combination with acute postoperative pain [19]. Buprenorphine is generally used to treat OUD, but it is a promising alternative to standard opioids for patients who both have concurrent chronic pain and an OUD or high-risk opioid use behavior. The findings of the present study support the use of buprenorphine/naloxone as a safe and efficient component of comprehensive pain therapy in these patients, with the benefit of close to six years of monitoring [20].

The incidence of buprenorphine misuse without OUD remained steady from 2015 to 2019 despite recent increases in buprenorphine treatment for OUD, while the prevalence of buprenorphine misuse with OUD trended lower. In 2019, misuse of oxycodone and hydrocodone was substantially more prevalent (by 4.9 million and 3.0 million adults, respectively). Contrarily, among the 2.4 million US adults who reported using buprenorphine in the past year, 0.7 million (or 29.2%) misused it. In the US, from 2015 to 2019, there was a decline in the proportion of persons using buprenorphine who also had an opioid use problem. Our findings highlight the need to take steps to increase access to buprenorphine-based OUD treatment, to create strategies to monitor and lessen the misuse of buprenorphine, and to address conditions like chronic pain, suicide risk, co-occurring mental illness, and polysubstance use that are related to misuse [21].

Some areas to fill in this field could be to explore different patients’ outcomes from buprenorphine other than pain ratings. There could be research focused on the frequency of side effects of buprenorphine when compared to other opioids and which drugs may be more indicated for certain populations based on side effects. There is an opportunity to expand this topic or conduct additional research on the long-term effects of buprenorphine when administered in different ways (IV, transdermal, sublingual, etc.). Further research can also be considered on how effective buprenorphine is for other types of surgeries such as cardiac surgeries, neurologic surgeries, and lung procedures.

Limitations

Despite a thorough review of the literature, we mostly found small trials with "good" outcomes for the outcome "duration of analgesia." Despite analyzing various studies, there were limitations to these studies. For example, all of the studies did not account for chronic conditions. Most accounted for age and gender, but each study had its own variation. In addition, not all studies were in the same age range group and did not come from the same location.

## Conclusions

Overall, keeping patients who were started on buprenorphine during surgery reduces postoperative problems. When used in pre-, peri-, and postoperative circumstances, buprenorphine provides various pain-management benefits and patients can still benefit from effective pain management from mu-opioid agonists while remaining on buprenorphine. Buprenorphine can be continued at a reduced dose as needed to avoid withdrawal symptoms and to improve the analgesic efficiency of mu-opioid agonists used in combination for acute postoperative pain considering the evidence at hand. Buprenorphine is also an attractive alternative to standard opioids for patients with chronic pain and an OUD. Buprenorphine administration needs a patient-centered, multidisciplinary strategy that considers the benefits and drawbacks of the many perioperative therapy options to have the best chance of success.
